# Leptomeningeal Carcinomatosis in Epithelial Ovarian Cancer: A Diagnostic Challenge

**DOI:** 10.7759/cureus.14440

**Published:** 2021-04-12

**Authors:** Nayha Tahir, Aatma Ram, Nikita Jain, Lalitha Padmanabha Vemireddy, Farah Zahra

**Affiliations:** 1 Internal Medicine, Chicago Medical School, Rosalind Franklin University of Medicine and Science, McHenry, USA

**Keywords:** leptomeningeal carcinomatosis, cerebrospinal fluid (csf), lumbar puncture (lp), ovarian cancers, intrathecal methotrexate, immunotherapy

## Abstract

Leptomeningeal carcinomatosis (LCM), also known as neoplastic meningitis, is a rare entity. It is generally seen in solid tumors. Ovarian cancers can infrequently cause LCM. The clinical presentation is variable. Diagnosis is made by a lumbar puncture that shows malignant cells in the cerebrospinal fluid (CSF) and usually correlates with imaging findings. Given the low individual sensitivities of lumbar puncture (55%) and magnetic resonance imaging (70%), it is recommended to combine both modalities for optimal diagnostic results. Treatment options vary depending on the type of primary carcinoma, however, the prognosis is guarded. We report a case of LCM in a patient with stage IV epithelial ovarian cancer in remission, which became a diagnostic challenge due to a lack of imaging findings.

## Introduction

Ovarian cancer (OC) ranks fifth among cancer-related deaths in American women and carries the highest mortality among all gynecological malignancies [1]. Epithelial-derived ovarian tumors are the most common type and usually present at advanced stages with an overall five-year survival <50% for stages III and IV [2]. The usual route of spread is a local invasion, with rare instances of hematogenous spread to solid organs [3]. Leptomeningeal carcinomatosis (LCM) or neoplastic meningitis is a rare complication of cancer that occurs in less than 2% of the cases where malignant cells infiltrate the cerebrospinal fluid (CSF) via leptomeninges - the arachnoid mater and pia mater. It is seen with some solid tumors, such as breast and lung carcinomas, however, LCM is extremely rare in cases of ovarian cancer [4]. We describe a case of a woman with stage IV epithelial ovarian cancer who developed LCM while being in remission with a negative imaging workup, making the case a diagnostic challenge.

## Case presentation

A 58-year-old woman, with a past medical history of stage IV epithelial OC presented to the emergency department (ED) with complaints of intermittent headaches of variable intensity and character for one month. Aggravating factors included bright light and loud noises while the headache was relieved with rest, dim light, and analgesics such as nonsteroidal anti-inflammatory agents. The headaches were associated with neck pain, nausea, episodic vomiting, blurry vision, and few episodes of lightheadedness. There was no history of fever, chills, gait instability, seizure activity, motor or sensory weakness, or loss of consciousness; however, the patient reported that a day prior to this admission, she had a syncopal episode. The syncopal episode happened while the patient was getting a neck massage by her daughter. The syncopal episode was not preceded by any warmth, flushed feeling, or sweating; it only lasted 30 seconds and the patient denied any post-syncopal confusion. Prior to this visit, she had been undergoing an extensive evaluation for these recurrent headaches during two additional ED visits and an outpatient neurology referral. Her past medical history was unremarkable except for the history of stage IV epithelial ovarian cancer, which was diagnosed nine months ago and was treated with six cycles of dense dose carboplatin and paclitaxel leading to remission with the latest CA 125 level of 32 U/ml.

**Figure 1:**
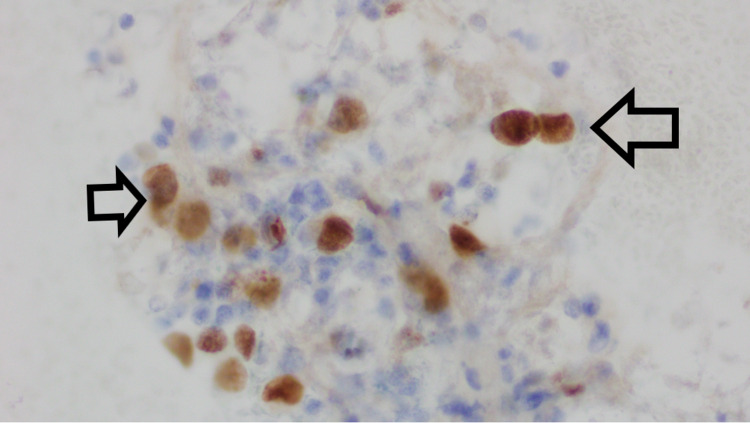
Group of atypical epithelial cells, with high nuclear to cytoplasmic ratio, and vacuolated, abundant cytoplasm The nuclei have fine chromatin, with prominent nucleoli (leftward arrow). There is atypical mitosis at the upper end of the cells (rightward arrow). PAX-8 immunostain shows strong, diffuse nuclear staining of the malignant cells, favoring adenocarcinoma of gynecological primary origin.

**Figure 2:**
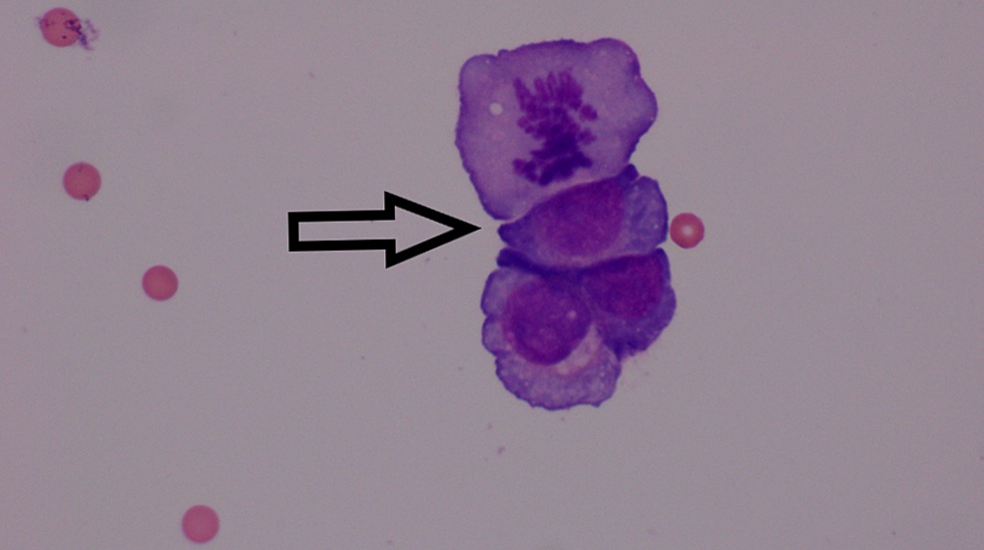
Eosin and methylene blue (E&M) stain showing atypical epithelial cells (arrow), with a high nuclear to cytoplasmic ratio, prominent nucleoli and abundant cytoplasm in the CSF fluid concerning for central nervous system metastasis CSF: cerebrospinal fluid

On physical examination, vital signs were normal (temperature of 98.5 °F (36.9 °C), oral), blood pressure of 155/72 mmHg, pulse rate of 75 beats per minute, respiratory rate of 20 breaths per minute, oxygen saturation of 100% on room air, and body mass index (BMI) 25.08 kg/m²]. The patient was alert, attentive, and oriented to time and person and did not exhibit any signs of scalp tenderness, nuchal rigidity, or meningismus. Her cranial nerve examination was intact, as were her strength, sensation, and reflexes throughout her extremities. There were no abnormal cerebellar findings and the rest of the physical examination was also normal. Initial laboratory work showed a white cell count of 10.1 K/µL, hemoglobin of 13.1 g/dl, platelets of 282 K/µL, sodium level of 138 mmol/L, potassium of 3.4 mmol/L, chloride of 107 mmol/L, blood urea nitrogen of 15 mg/dl, creatinine of 0.68 mg/dl, blood glucose 113 mg/dl, and creatine kinase of 89 u/L. Urinalysis was unremarkable. Polymerase chain reaction (PCR) by nasopharyngeal swab for severe acute respiratory syndrome coronavirus 2 (SARS-CoV-2) was negative. Troponin was < 0.04 ng/ml. An electrocardiogram, as well as an echocardiogram, were unremarkable. An extensive radiological evaluation during multiple ED and outpatient visits included a computed tomography (CT) angiogram of the head and neck, computed tomography (CT) venogram brain, magnetic resonance imaging (MRI) brain and cervical cord, CT lumbar spine, and CT abdomen and pelvis, all of which were unremarkable. Given headaches were intermittent and associated with photophobia, nausea, and vomiting, a diagnosis of migraine was initially considered, and the patient received symptomatic treatment outpatient. However, given the recent episode of unexplained syncope and a past medical history of cancer, the patient was admitted to rule out possible causes of syncope. The hospital course was complicated by an episode of a generalized tonic-clonic seizure lasting one minute. The patient was started on levetiracetam along with empirical antibiotics, including ceftriaxone and vancomycin, given concerns for meningitis. A neurology consultation was requested, who recommended a lumbar puncture. Infectious meningitis was ruled out given a bland CSF analysis. CSF cytology also came back negative for any malignant cells. Despite all the workup being negative, the patient's symptoms remained persistent. The underlying history of cancer at this point prompted a second lumbar puncture performed 24 hours apart from the first one. Repeat CSF cytology showed malignant-appearing cells consistent with metastatic adenocarcinoma of primary gynecological origin consistent with the diagnosis of LCM (Figures 1-2).

Given the above-mentioned findings in CSF cytology, an Ommaya catheter was immediately placed for the prompt initiation of intrathecal methotrexate chemotherapy. However, 24 hours postoperatively, the patient started deteriorating, with worsening headaches and frequent seizures, and the family decided to opt for hospice care. The patient was kept comfortable on intravenous morphine and supplemental oxygen until she passed away the next morning.

## Discussion

OC is the leading cause of gynecologic cancer deaths among American women [5]. In 2021, OC alone is estimated to cause more than 21,000 new cases and more than 13,000 deaths in the U.S [5]. Due to a lack of screening tests and an asymptomatic presentation during early stages, most patients are diagnosed at advanced stages (III and IV). OC primarily spreads locally, involving adjacent pelvic organs and/or overlying peritoneum [6]. Distant metastasis is less common and can involve the liver, lymph nodes, lungs, bones, and brain in decreasing frequency [7].

LCM is a dreadful complication and has been reported in many cancers. Incidence varies by cancer type and is overall estimated at 5%-10% in all solid tumors, with melanoma, breast cancer, and lung cancer being the most common culprits [8-9]. In recent years, LCM incidence has been rising, presumably due to improved imaging techniques and improved antineoplastic therapies leading to improved overall survival in various cancers [10]. In contrast, LCM incidence in ovarian cancer is exceedingly rare and usually occurs in the setting of disseminated disease [7]. Isolated leptomeningeal metastasis in ovarian cancer is extremely uncommon and described in the literature mostly in case reports and case series [7].

We describe a rare case of epithelial ovarian cancer, in remission, with a recent negative follow-up positron emission tomography (PET) scan and cancer antigen (CA) 125 level of 32 U/ml, who presented to the hospital with nonspecific neurological symptoms. Extensive workup demonstrated no apparent parenchymal brain lesions and no significant imaging findings elsewhere. The diagnosis was made based on repeat CSF analysis revealing solitary malignant appearing cells with strong PAX 8 positivity on immunohistochemistry, consistent with metastatic adenocarcinoma of gynecological origin.

Through this case, we want to highlight the importance of the prompt initiation of the diagnostic workup in a cancer patient presenting with neurological symptoms and the importance of repeat testing in appropriate clinical settings. Presenting symptomatology can be nonspecific and may be overlooked; a high index of suspicion is required in these patients to evaluate for LCM. Clinical presentation varies depending upon the site of involvement and can broadly be categorized into the features of cranial or spinal nerve dysfunction, raised intracranial pressure, focal neurological deficits, and meningeal irritation [10].

Evaluation begins with a history and thorough neurological examination. MRI brain and spine with contrast should be performed in cases with high suspicion of LCM and to rule out other infectious, inflammatory, and parenchymal lesions as potential causes. The diagnosis can be challenging without a definitive test to rule out meningeal involvement [11]. Positive MRI brain and spine with contrast findings in a correct clinical setting are sufficient to make a diagnosis of LCM. However, due to low sensitivity (70%), a negative MRI brain and spine with contrast does not exclude LCM. In most cases, lumbar puncture is recommended, and CSF cytology in conjunction with imaging remains the gold standard [10]. A repeat lumbar puncture may be necessary to increase diagnostic yield, increasing sensitivity to as high as 95% at three high-volume attempts (10 ml) lumbar punctures [10].

Despite the rigorous workup necessary to diagnose LCM, the prognosis is poor. Median survival after diagnosis is estimated at six to eight weeks without tumor-specific treatment and up to a few months with tumor-specific treatment [9]. Systemic chemotherapy, radiotherapy, and intrathecal pharmacotherapy alone or in combination remain the mainstay of treatment. A chemotherapeutic agent is chosen based on the primary cancer type and chemosensitivity of primary cancer when available. Surgical intervention can be considered for symptom control in diffuse leptomeningeal involvement and in the setting of solitary parenchymal or leptomeningeal lesions [11]. In this modern era, the use of targeted therapies to treat LCM in non-small cell lung cancer has shown survival benefits with better safety profiles [12]. A recent single-arm, open-label phase two trial of pembrolizumab in patients of solid tumor malignancies with leptomeningeal carcinomatosis was also published in June 2020. It showed some improvement in three-month survival, albeit with a small sample size [13]. Further research is needed to investigate similar treatment options for LCM in ovarian cancer. In patients with poor prognosis, the prompt initiation of goals of care discussions and comfort measures can be a reasonable option to improve quality of life.

## Conclusions

A high index of suspicion is warranted to rule out LCM in a cancer patient presenting with unexplained neurological symptoms. During the evaluation process, physicians may need to rely on repetitive and extensive diagnostic modalities to avoid misdiagnosis. Negative CSF analysis should not rule out the diagnosis in highly suspicious cases; repeat LP is recommended in the appropriate clinical scenarios. An increasing number of patients with solid tumor malignancies have been found to have LCM given improved diagnostic and therapeutic options, but survival among LCM patients remains poor. More studies are needed to investigate further the role of immunotherapy in improving survival among LCM patients diagnosed with ovarian cancer.
